# Assessing the Contributions of Inactivation, Removal, and Transfer of Ebola Virus and Vesicular Stomatitis Virus by Disinfectant Pre-soaked Wipes

**DOI:** 10.3389/fpubh.2020.00183

**Published:** 2020-06-02

**Authors:** Todd A. Cutts, Catherine Robertson, Steven S. Theriault, Raymond W. Nims, Samantha B. Kasloff, Joseph R. Rubino, M. Khalid Ijaz

**Affiliations:** ^1^Canadian Science Centre for Human and Animal Health, Winnipeg, MB, Canada; ^2^J.C. Wilt Infectious Diseases Research Centre, Public Health Agency of Canada, Winnipeg, MB, Canada; ^3^Department of Microbiology, The University of Manitoba, Winnipeg, MB, Canada; ^4^RMC Pharmaceutical Solutions, Inc., Longmont, CO, United States; ^5^Reckitt Benckiser LLC, Global Research & Development for Lysol and Dettol, Montvale, NJ, United States; ^6^Department of Biology, Medgar Evers College of the City University of New York (CUNY), Brooklyn, NY, United States

**Keywords:** ASTM E2967-15, Disinfectant pre-soaked wipes, Ebola virus—Makona strain, inactivation, removal, transfer, vesicular stomatitis virus, wiperator

## Abstract

Disinfectant pre-soaked wipes (DPW) containing activated hydrogen peroxide (AHP) or quaternary ammonium compounds (QAC) were tested using ASTM E2967-15 to determine removal, transfer, and inactivation of Ebola virus Makona variant (EBOV/Mak) and vesicular stomatitis virus (VSV) from contaminated stainless steel prototypic environmental surfaces. The infectious virus-contaminated carriers were subjected to wiping in the Wiperator per the standard. Following the use of negative control (J-Cloth)-, AHP-, or QAC-based wipes, recovery of residual infectious virus was assayed. In the case of the J-Cloth wipes (negative control), although removal of virus from inoculated carriers was extensive i.e., ~99% (1.9–3.5 log_10_) transfer of virus by these wipes to a secondary surface amounted to ≤ 2% (~3.8 log_10_) of the initial virus load. In the case of each DPW, >6 log_10_ removal/inactivation of virus was observed, with limited (EBOV/Mak) or no (VSV) virus transfer observed. The efficacy of wipes for decontaminating high-touch environmental surfaces spiked with EBOV/Mak or VSV is discussed. In summary, removal of EBOV/Mak and VSV using wipes was extensive in this study. In the absence of a sufficient concentration and contact time of an appropriate microbicidal active in DPW (such as the AHP- and QAC-based DPW tested), transfer of a low, albeit significant (from an infectious unit/infectious dose perspective), quantity of infectious virus from the inoculated surface to a secondary surface was observed. In the case of Ebola virus, it is essential that a DPW with an appropriate microbicidal active, following the appropriate contact time, be used to prevent unintended transfer of infectious virus to a clean secondary surface (as observed in negative control /J-Cloth). Otherwise, there exists the possibility of dissemination of Ebola virus and the associated risk of transmission of Ebola virus disease.

## Introduction

Disinfectant pre-soaked wipes (DPW) are increasingly being used to disinfect high-touch environmental surfaces (HITES) in healthcare and home settings (1, 2). In the case of emerging viruses, the use of DPW as an intervention in the cycle of infection transmission can be effective as a result of two orthogonal mechanisms of reducing viral load on contaminated HITES (1), viral inactivation (reduction of infectious virus) and physical removal of the virus from the HITES by wiping. The term *decontamination* has been proposed by Sattar and Maillard (3) to include both the removal and the inactivation functions of such wipes. A wipe which only provides physical removal of a pathogen actually represents a source of infectious agent-dissemination by transfer of the pathogen from the contaminated surface to a non-contaminated surface. This could potentially lead to spreading of the infectious agent from the original source to new surfaces (3–5). For this reason, wipes that do not contain an appropriate microbicidal active may not represent a useful intervention for limiting the spread of infectious disease caused by pathogens contaminating environmental surfaces. How significant is the potential for pathogen spread using such wipes?

Until recently, it has not been practically possible to evaluate separately the efficacies of DPW for removal vs. inactivation of pathogens, and of the potential for transfer of removed pathogens including viruses picked up during wiping to other surfaces. The recent development and use of a standard testing method [ASTM E2967-15 (6)], utilizing an instrument known as the Wiperator (FiltaFlex, Almonte, ON) (6–8), has allowed investigators to parse out the contributions of inactivation and removal toward overall effectiveness of DPW for bacterial pathogens such as *Staphylococcus aureus* (7, 8), *Acinetobacter baumannii* (7, 8), and *Clostridium difficile* (7). More recently, such investigations have been extended to viral pathogens, as well. For instance, Wesgate and Maillard (9) evaluated transfer and inactivation of the bacteriophage MS2 using the Wiperator. Becker et al. (10) evaluated a variety of DPW for efficacy against three non-enveloped viruses (murine norovirus, human adenovirus type 5, and SV40 virus) using the 4-field test [EN 16615:2015 (11)]. The latter represents an alternative methodology for evaluating removal vs. inactivation for DPW.

In the present paper, we have investigated the efficacy of DPW for removal, inactivation, and transfer of Ebola virus and of vesicular stomatitis virus (VSV) from stainless steel surfaces. Our interest in this study was to evaluate two enveloped viruses, each of which was expected to be inactivated by the microbicidal active used in the DPW, while each test virus was expected to be removed, but not inactivated, by the control wipe without microbicidal active. In addition, VSV has been used as a surrogate for the Ebola virus in previous studies (12). The two viruses differ, of course, in one very specific attribute, and that is their lethality to humans. That is, VSV infections in humans lead to very mild symptomology, while Ebola infections are associated with high mortality (13, 14). Ebola virus is a relatively large (80 × 14000 nm), enveloped, cylindrical shaped, negative single-stranded RNA virus of the *Filoviridae* family (14). Vesicular stomatitis virus has been used as surrogate virus for studying Ebola virus inactivation, since VSV (70 × 170 nm) is also a relatively large, enveloped, bullet-shaped, negative single-stranded RNA virus, in this case from the *Rhabdoviridae* virus family (15). The results for two different microbicidal active-containing DPW have been compared to results obtained with a wipe containing DMEM only (no active ingredient).

## Materials and Methods

### Cell Line, Viruses, and Medium

African green monkey Vero E6 cells (ATCC CRL-1586; American Type Culture Collection, Manassas, VA, United States) were maintained at 37°C/5% CO_2_ in Dulbecco's modified Eagle medium (DMEM; HyClone, Logan, UT, United States) supplemented with 10% fetal bovine serum (FBS; Gibco, Grand Island, NY, United States) and 10 units/ml penicillin/streptomycin (Gibco). Ebola virus Makona variant (EBOV/Mak; Ebola virus/H. sapiens-tc/GIN/2014/Makona-C05; GenBank accession no. KJ660348) was obtained from a clinical isolate and biotechnologically engineered to express green fluorescent protein (GFP). Vesicular stomatitis virus (VSV) stocks were prepared from a reverse genetics construct (16, 17) and biotechnologically engineered to express GFP.

### Stock Virus Preparation

**Figure 1:**
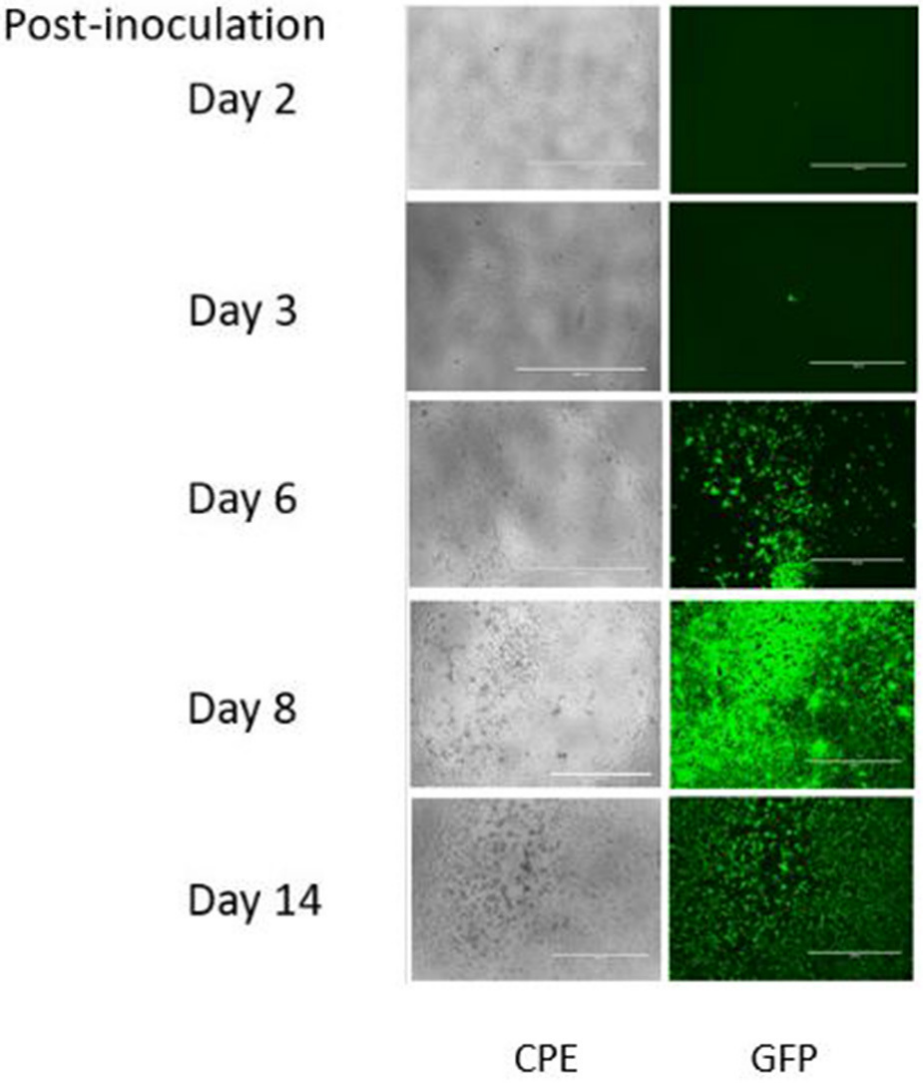
Appearance of viral CPE or GFP in Vero E6 cells 2–14 days post-inoculation with EBOV/Mak.

A characterized stock of EBOV/Mak virus was prepared by infecting five T-175 flasks of Vero E6 cells with EBOV/Mak virus expressing GFP at a multiplicity of infection of 0.01. Vero E6 cells appear to be a superior host cell for amplifying and detecting Ebola virus and are indicated in the American Type Culture Collection cell description (18) as susceptible to a number of hemorrhagic disease viruses, including Zaire Ebola virus. The cell line is also susceptible to infection by VSV used in this investigation. Expression of the GFP was evident in the infected Vero E6 cells in as little as 3 days post-inoculation, although viral cytopathic effect (CPE), defined as cellular detachment, degeneration of the cell sheet, and cell rounding was not observed until ~6 days post-inoculation (Figure 1). At this time, the flasks were frozen at −70°C. The flasks were thawed the following day and the conditioned medium was removed and clarified by low-speed centrifugation (4500 × g) for 10 min. The clarified supernatant from each flask was pooled and overlaid onto a 20% w/v sucrose cushion in Tris-NaCl-EDTA (TNE) buffer. The centrifuge tubes were spun at 134,000 RCF Max (Beckman Coulter 30 Ti rotor at 28,000 rpm) for 2 h within a SW 32 Ti Rotor. The supernatants were discarded and the remaining viral pellets were resuspended in DMEM + 2% FBS + 10 units/ml penicillin/streptomycin (VCM) overnight at 4°C. Virus stocks were pooled, aliquoted into usable amounts, and frozen at −70°C until needed. Working stock virus titers were determined, on the basis of viral CPE/GFP in Vero E6 cells, to be in excess of 8.8 log_10_ TCID_50_/ml by the Reed-Muench procedure (19). As demonstrated in Figure 1, the use of GFP as an indicator of infection greatly enhances the sensitivity of the assay by having an earlier readout with ability to visualize a positive outcome in a smaller percentage of the detector cells. In addition, the GFP readout is considered more specific, as apparent CPE may result from other factors such as cytotoxicity or prolonged incubation periods leading to overconfluence and cell sloughing.

A stock cryovial of VSV strain Indiana was thawed and used to infect a nearly confluent T-175 flask of Vero E6 cells. Briefly, cell culture medium (DMEM + 10% FBS + 10 units/ml penicillin/streptomycin [CCM]) was aspirated from the T-175 flask and replaced with 5 ml of diluted virus stock (MOI 0.01) overlaid for 1 h at 37°C ± 5% CO_2_. The flask was gently rocked every 10 min over 1 h. Afterwards, the inoculum was aspirated and replaced with 15 ml of fresh VCM and incubated for 72 h, until the majority of the cell monolayer was dislodged. The flask was frozen at −70°C overnight and subsequently thawed the following day. The conditioned medium was clarified by low-speed centrifugation (5000 × g for 10 min) and the supernatant was aliquoted and stored at −70°C until needed. Working stock virus titers were determined, on the basis of viral CPE in Vero E6 cells, to be in excess of 8.8 log_10_ TCID_50_/ml by the Reed-Muench procedure (19).

### Negative Control Wipes

The “J-Cloth,” a representative cloth material that has been used in previous studies of this type (6, 8) was used for negative controls. The J-Cloth material was cut into 4 cm × 4 cm squares and the squares were autoclaved at 121°C for 60 min. Using sterile forceps, a single sterile J-Cloth square was placed into a plastic Petri dish and 320 μl of DMEM were added to the square. Saturated J-Cloth squares were placed onto the Wiperator boss per the ASTM 2967-15 standard (6).

### Preparation of Wipes for Efficacy Assay

**Figure 2:**
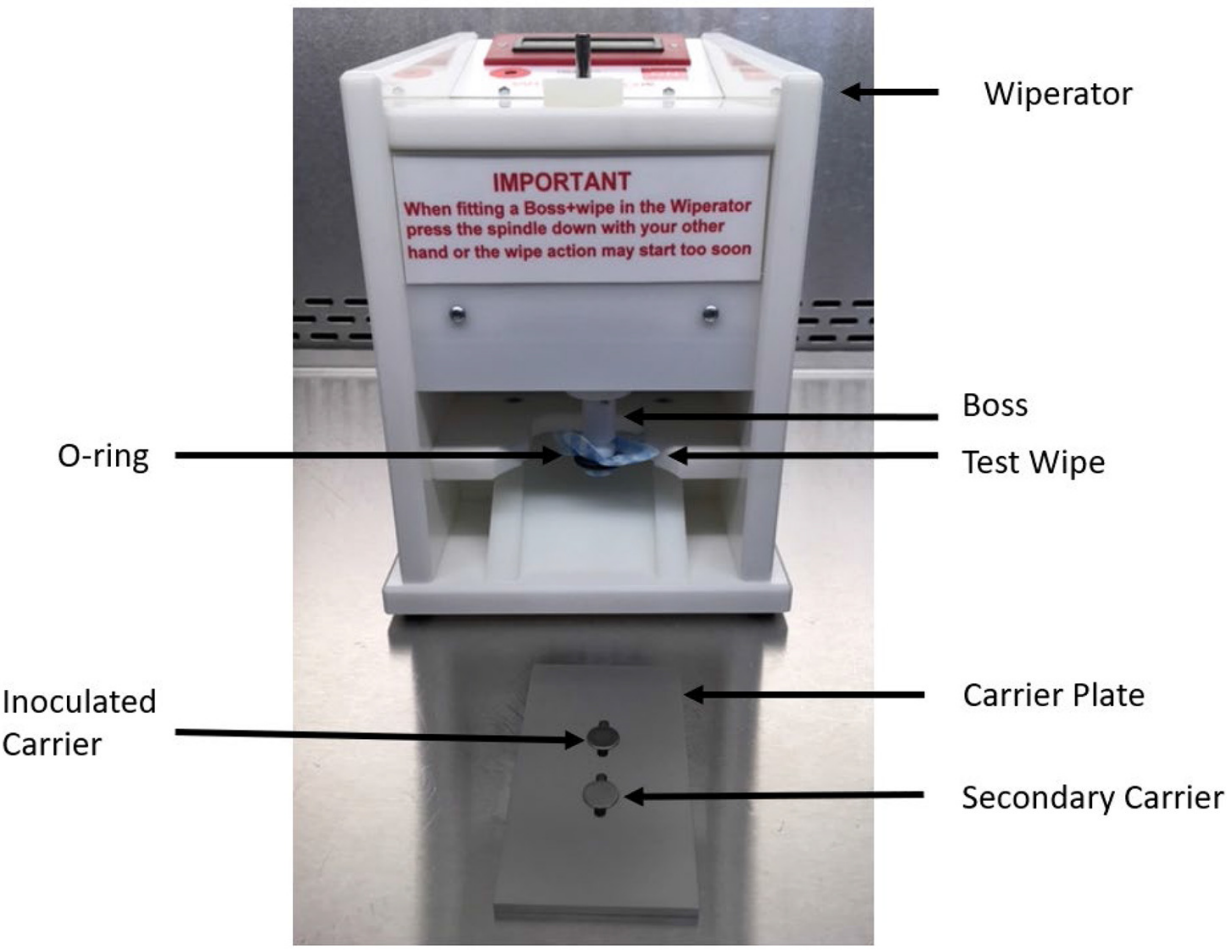
Components of the Wiperator used to investigate removal, transfer, and inactivation of VSV and EBOV/Mak by DPW and control wipes.

“AHP” wipes consisted of J-Cloth squares impregnated with a 1:40 solution of accelerated hydrogen peroxide (AHP). Sterile J-Cloths (4 × 4 cm) were prepared in the same manner as mentioned above. A single sterile J-Cloth was placed into a sterile Petri plate with 320 μl of prepared AHP added to the wipe. The impregnated wipe was immediately placed overtop the sterile O-ring (Figure 2) and, a sterile Wiperator Boss (the part of the instrument which manipulates the test wipe) was pushed into the wipe (8). The loaded wipe was added to the Wiperator where it was used to wipe pre-inoculated surfaces and then used to wipe a secondary (non-inoculated) surface.

“QAC” wipes consisted of a ready-to-use commercially available wipe impregnated with a quaternary ammonium compound (QAC: benzyl-C12-16-alkyldimethyl chloride; three independent lots). Each of the QAC wipe lots examined were tested at the end of their stated expiry dates. The QAC wipes were used fresh on the day of each assay. Prior to use in an assay, a container containing QAC wipes was inverted for 10 s five times. Using sterile forceps, three QAC wipes were removed and deposited into a sterile plastic Petri dish (150 × 15 mm). Additional QAC wipes were removed from the same container and sixteen 4 × 4 cm squares were excised from the center of four QAC wipes using sterilized forceps and scissors. Several other QAC wipes were removed from the QAC wipe container and overlaid onto the excised squares to keep the test squares moist. A plastic lid was placed on top of the Petri dish, and the dish was sealed with Parafilm and transported to the biosafety hood in the testing laboratory. Prior to efficacy testing, sealed Petri dishes were inverted as described above and the wipe squares were removed using sterile forceps and loaded onto the Wiperator Boss (8). Excised wipe samples were added to the Boss within 2 h after being sealed into the Petri dish.

### Disinfectant Neutralization Assay

Prior to evaluating the efficacy of wipes for decontaminating infectious virus (Ebola virus or VSV), a neutralization assay was performed to evaluate the interaction of neutralizers and disinfectants, cytotoxicity to the Vero E6 cells used for reporting GFP/CPE, and impacts on the recovery of infectious virus from the surfaces post-test. Combinations of neutralizers, disinfectants, and viruses were evaluated.

On the day of neutralization assay, several neutralizing reagents (VCM, 1 × Letheen Broth, and 1 × Letheen Broth + VCM [1:10 10 × Letheen broth stock combined with VCM]) were prepared fresh. In addition, a low titer virus stock (~100–1000 TCID_50_ per mL) of VSV or EBOV/Mak was also prepared with only 10 μl inoculum used for the appropriate test conditions requiring live virus. All neutralization controls were performed in replicates of three over a single experiment. Neutralization controls included the following:

#### Negative Control

Cells were cultured in VCM and used as a control for evaluation of cytotoxicity and viral CPE.

#### Neutralizer Control

The neutralizer being evaluated was ten-fold serially diluted in VCM and 50 μl were added to Vero E6 cells in replicates of five for each dilution from 10^0^ (neat) to 10^−3^. Cells were scored for cytotoxicity 14 days post-inoculation.

#### Neutralizer Disinfectant (Cytotoxicity Control)

Microbicidal active-impregnated wipes or DMEM impregnated wipes were loaded onto the Wiperator and a sterile stainless steel coupon was exposed to the wipe for 5 s. The exposed stainless steel coupon was placed into 1 ml of neutralizer, mixed by pipetting, and finally 10-fold serially diluted in VCM (10^0^ to 10^−3^). For each dilution, 50 μl were added to an 80% confluent Vero E6 cell monolayer in replicates of five and monitored over 14 days for CPE. Three technical replicates were used for each test wipe.

#### Positive Control (Virus)

The positive virus control was prepared by adding 10 μl of diluted low titer virus to 990 μl of VCM. The positive control was 10-fold serially diluted in VCM and 50 μl of each dilution were added to Vero E6 cells in replicates of five for each dilution from 10^0^ (neat) to 10^−3^. Cells were scored for viral CPE 14 days post-inoculation.

#### Neutralizer Virus Control (Virus)

To account for the effect of the neutralizer acting on the virus, 10 μl of low titer virus were added to 990 μl of neutralizer. The neutralizer virus control samples were 10-fold serially diluted in VCM and 50 μl were added to Vero E6 cells in replicates of five for each dilution from 10^0^ (neat) to 10^−3^. Cells were scored for viral CPE 14 days post-inoculation.

#### Neutralizer Disinfectant Virus Control (Virus)

To account for the ability for the neutralizer to affectively mitigate the effects of the disinfectant active tested, a prepared active impregnated wipe was loaded onto the Wiperator and the sterile stainless steel carrier was exposed to the active wipe for 5 s. The “wiped” stainless steel carrier was placed into 1 ml of neutralizer, the liquid was mixed by pipetting, and 10 μl of low-titer virus were added. The neutralization disinfectant virus control sample was incubated for 10 min at room temperature after which it was 10-fold serially diluted in VCM with 50 μl added in replicates of 5 added to Vero E6 cells for each dilution from 10^0^ (neat) to 10^−3^. Cells were scored for viral CPE 14 days post-inoculation.

### Efficacy Testing of Wipes

**Figure 3:**
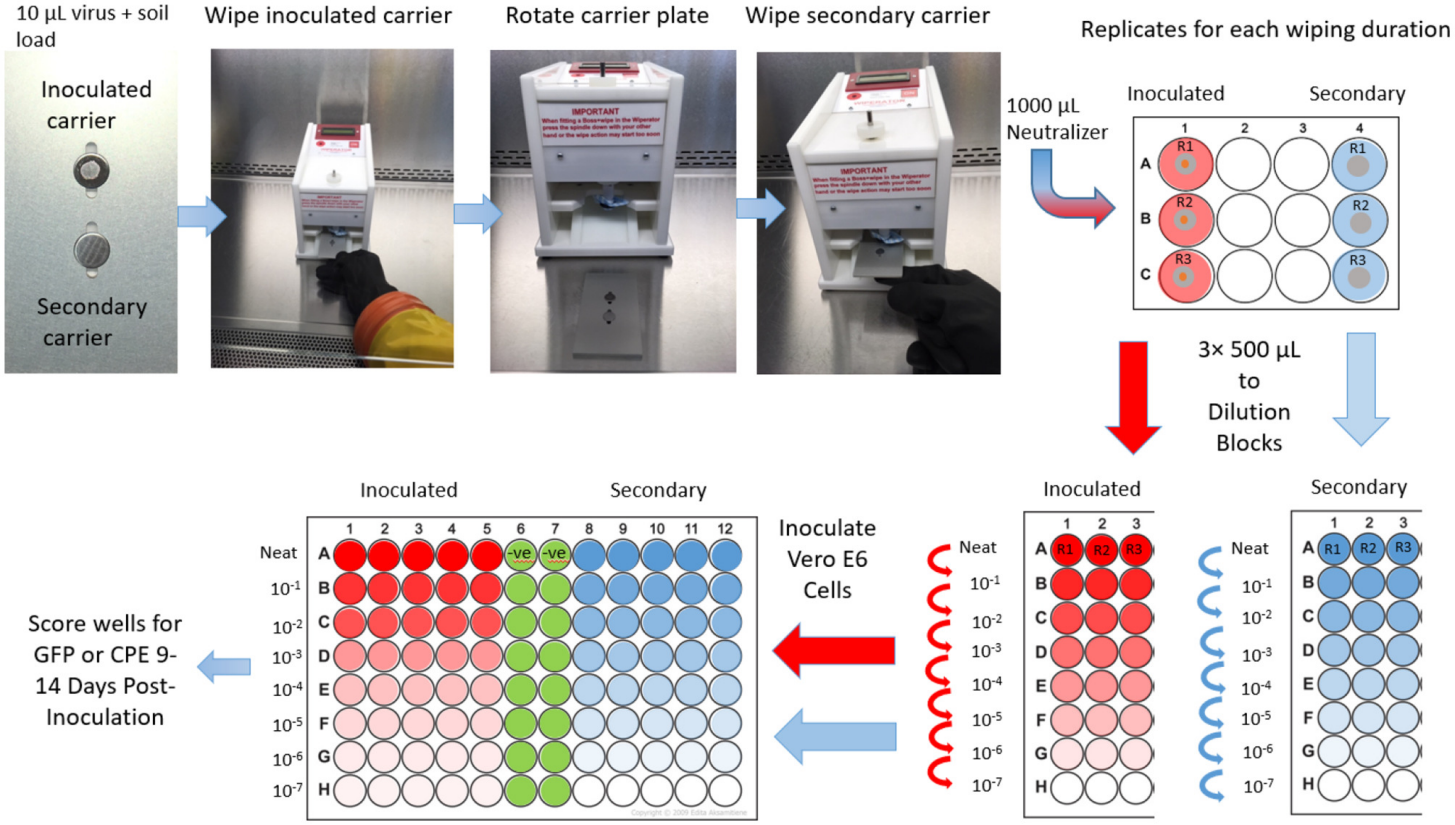
Schematic representation of the inactivation/removal testing methodology employed.

Efficacy testing of wipes (Figure 3) was performed per ASTM 2967-15 (6). Test virus inocula were prepared in a tripartite soil load (20, 21). A “soil load” refers to a matrix designed to challenge the test virus inactivation and removal process. The term “soil load” is sometimes replaced with the term “organic load” and the latter may be more descriptive of the typical challenge matrix, which is intended to simulate secretions or excretions in which the virus would be released from an infected person. In our study, we used the tripartite soil load specified in the ASTM standard, which consists of sterile components (12.5 μl of 5% bovine serum albumin + 17.5 μl 5% tryptone + 50 μl 0.4% mucin) to which was added 170 μl virus stock. This was prepared fresh daily for each replicate of the virucidal test performed. Using a positive displacement pipette, prepared virus (10 μl) was deposited onto sterile stainless steel carriers and the carriers were air-dried in a Class II BSC within a BSL-2 laboratory (VSV) or a BSL-4 laboratory (EBOV/Mak) for 60 min. Inoculated carriers were placed into one grove of the Wiperator carrier plate (Figure 2) and secured in place by use of a magnet on the opposite side of the plate. A second non-inoculated carrier was placed in a second fitted slot and secured with a magnet on the opposite side of the carrier plates.

Using sterile forceps, sterile microbicide-impregnated active wipes or sterile wipes impregnated with DMEM were removed from the Petri dishes, loaded onto the Wiperator Boss, and secured in place with a large O-ring (Figure 2). Loaded Bosses were then attached to the Wiperator spindle and the plates containing the carriers lifted into place. The orbital wiping action commenced as soon as contact was made with the plate. The orbiting parameters were 10 mm diameter, with 150 g of pressure for time points of 5, 15, 30, or 60 s wiping time. After the time points were reached, the carrier plate was dropped from the wiping position and flipped so a sterile non-inoculated carrier was below the used wipe and lifted into position. Orbiting action started as soon as contact was made with the secondary container and continued for 5 seconds wiping time per cycle at 150 g pressure. The plate containing the two carriers was then removed from the Wiperator and the exposed carriers retrieved using sterile forceps. Treated carriers were placed into 1 ml of VCM neutralizing solution and the infectious virus remaining on the carriers was recovered by pipetting.

## Results

### Neutralization Effectiveness Evaluation

**Figure 4:**
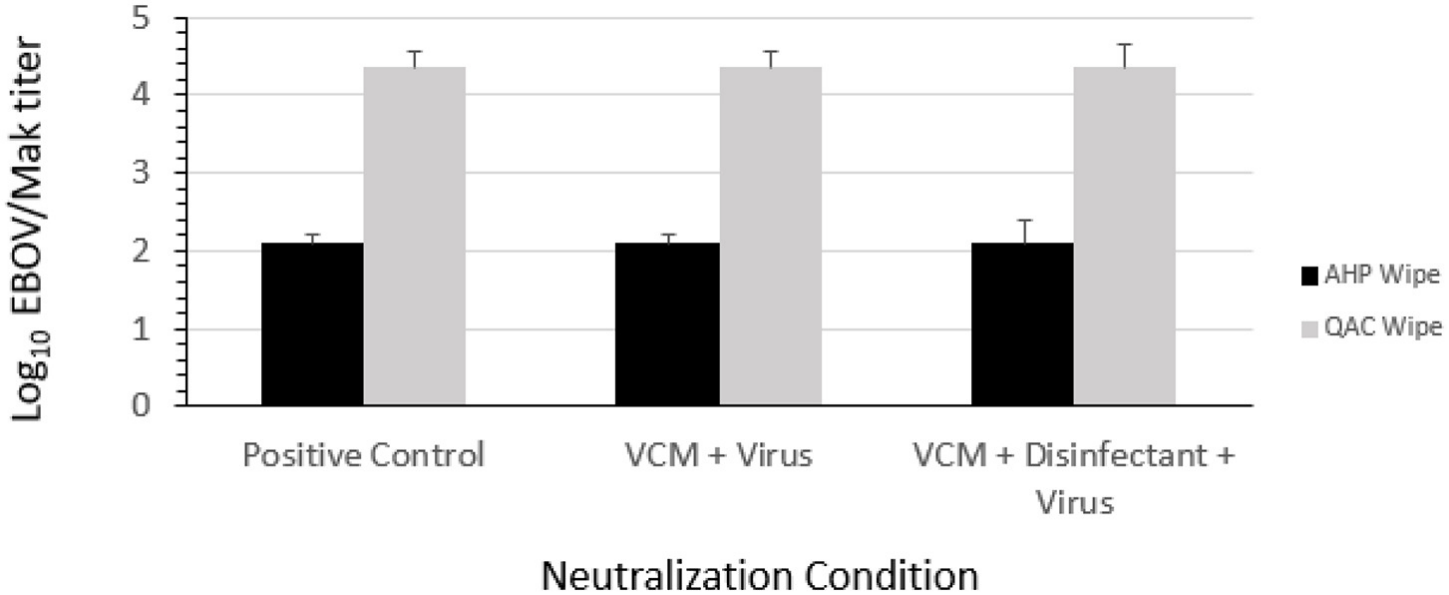
Ability of VCM to neutralize the EBOV/Mak-inactivating effects of QAC and AHP disinfectant from DPW wipes.

During the evaluation of possible neutralizing agents, it was determined that 100% virus culture medium (VCM) added to the AHP or QAC wipe dilutions prior to introduction of the EBOV/Mak or VSV in tripartite soil load (21) prevented inactivation of the viruses. As shown in Table 1, VCM (neutralizer) alone and VCM + disinfectant did not cause cytotoxicity to Vero E6 cells, even applied undiluted. As shown in Figure 4, the viral titers obtained for the virus positive controls, the neutralizer (VCM) + EBOV/Mak, and the neutralizer (VCM) + disinfectant + EBOV/Mak were indistinguishable. The disinfectant neutralizing agent that was used in each of the inactivation efficacy studies described below was VCM.

**Table 1:** Cytotoxicity evaluation for Vero E6 cells exposed to negative control (VCM neutralizer) or neutralizer + QAC or AHP disinfectant from DPW wipe.

**Test Condition**	**QAC wipe replicates**			**AHP wipe replicates**		
	**1**	**2**	**3**	**1**	**2**	**3**
**Negative control (VCM)**	10^0^	10^0^	10^0^	10^0^	10^0^	10^0^
**Neutralizer** **+** **Disinfectant**	10^0^	10^0^	10^0^	10^0^	10^0^	10^0^

*The lowest dilution of the post-neutralization solution which did not cause cytotoxicity to the Vero-E6 cells is shown (10^0^ = undiluted)*.

### Removal and Inactivation Efficacy Testing

The results obtained in carrier testing of a single lot of AHP-impregnated wipes and three lots of QAC-impregnated wipes and of the negative control (J-Cloth) wipes are displayed in Table 2 (EBOV/Mak) and Table 3 (VSV). In the absence of expected viral inactivation (i.e., in the case of the negative control wipe), extent of virus *removal* was estimated by the difference in titer of infectious virus recovered from the dried positive control carrier and that recovered from the inoculated carrier following use of the wipe by the Wiperator at the various wiping times evaluated. Extent of virus *transfer* was expressed as a percentage of the infectious virus estimated (on the basis of recovery from the dried positive control condition) to be on the inoculated control carrier that was recovered from the secondary carrier after the 5-s transfer wiping step. The amount of virus remaining on the wipe itself was not measured, therefore complete mass balances for the spiking viruses were not calculable. As a result, *inactivation* could not be quantitatively measured, but was implied by the absence of viral recovery from the inoculated carriers following wiping for 5, 15, 30, or 60 s, or from the secondary carrier following the 5-s infectious virus transfer wiping step.

**Table 2:** Efficacy of disinfectant-impregnated wipes vs. DMEM-impregnated wipes for removal, transfer, and inactivation of EBOV-Mak^*^.

**Condition**	**Positive controls**		**EBOV-Mak titer (log**_****10****_ **TCID**_****50****_**/mL) after wiping time:**			
	**Initial**	**Dried**	**5 s**	**15 s**	**30 s**	**60 s**
**J-Cloth DMEM wipes**						
Inoculated carrier	6.75 ± 0.25	6.55 ± 0.28	4.09 ± 0.38	3.52 ± 0.80	3.17 ± 0.56	3.09 ± 0.69
Removal			~2.5 log_10_	~3.0 log_10_	~3.4 log_10_	~3.5 log_10_
Secondary carrier			3.94 ± 0.85	3.49 ± 0.63	2.91 ± 0.96	2.26 ± 0.70
Transfer			0.41%	0.13%	0.09%	0.01%
**AHP wipes**						
Inoculated carrier	6.80 ± 0	6.62 ± 0.30	1.14 ± 1.56	0.32 ± 0.66	0.51 ± 1.05	0.20 ± 0.60
Removal/Inactivation			~5.5 log_10_	~6.3 log_10_	~6.1 log_10_	~6.4 log_10_
Secondary carrier			0.93 ± 1.40	0	0.12 ± 0.35	0
Transfer			0.004%	0%	<0.0002%	0%
**QAC wipes**						
Inoculated carrier	6.90 ± 0.15	6.59 ± 0.27	0.58 ± 0.93	0	0.19 ± 0.56	0
Removal/Inactivation			~6.0 log_10_	~6.6 log_10_	~6.4 log_10_	~6.6 log_10_
Secondary carrier			0.20 ± 0.60	0	0	0
Transfer			0.0002%	0%	0%	0%

* *Values displayed are the log_10_ infectious virus titer in units of log_10_ TCID_50_/mL for positive controls and the results of Wiperator post-testing after various wiping times. The results are the mean ± standard deviation for n = 9 [3 replicates each in each of 3 assays]. Removal values shown for the J-Cloth wipe represent the mean log_10_ of the infectious virus load (TCID_50_) for the dried positive control carrier minus the mean log_10_ of the infectious virus recovered from the inoculated carrier. Transfer is expressed as the percent of the infectious virus recovered from the positive control dried carrier that is recovered from the secondary carrier*.

**Table 3:** Efficacy of disinfectant-impregnated wipes vs. DMEM-impregnated wipes for removal, transfer, and inactivation of VSV^*^.

**Condition**	**Positive controls**		**VSV titer (log**_****10****_ **TCID**_****50****_**/mL) after wiping time:**			
	**Initial**	**Dried**	**5 s**	**15 s**	**30 s**	**60 s**
**J-Cloth DMEM wipes**						
Inoculated carrier	6.80 ± 0.00	5.78 ± 0.33	3.85 ± 0.51	3.40 ± 0.41	3.46 ± 0.36	3.32 ± 0.40
Removal			~1.9 log_10_	~2.4 log_10_	~2.3 log_10_	~2.5 log_10_
Secondary carrier			3.79 ± 0.57	3.48 ± 0.39	3.26 ± 0.58	3.36 ± 0.34
Transfer			1.70%	0.54%	0.40%	0.39%
**AHP wipes**						
Inoculated carrier	7.43 ± 0.71	6.21 ± 1.07	0	0	0	0
Removal/Inactivation			~6.2 log_10_	~6.2 log_10_	~6.2 log_10_	~6.2 log_10_
Secondary carrier			0	0	0	0
**QAC wipes**						
Inoculated carrier	6.47	6.02 ± 0.19	0	0	0	0
Removal/Inactivation			~6.0 log_10_	~6.0 log_10_	~6.0 log_10_	~6.0 log_10_
Secondary carrier			0	0	0	0

* *Values displayed are the log_10_ infectious virus titer in units of log_10_ TCID_50_/mL for positive controls and the results of Wiperator post-testing after various wiping times. The results are the mean ± standard deviation for n = 9 [3 replicates each in each of 3 assays]. Removal values shown for the J-Cloth wipe represent the mean log_10_ of the infectious virus load (TCID_50_) for the dried positive control carrier minus the mean log_10_ of the infectious virus recovered from the inoculated carrier. Transfer is expressed as the percent of the infectious virus recovered from the positive control dried carrier that is recovered from the secondary carrier*.

### Results for EBOV/Mak

All testing involving EBOV/Mak was performed at room temperature within a Class II BSC in the BSL-4 laboratories of Public Health Agency of Canada at the Canadian Science Center for Human and Animal Health, Winnipeg, Manitoba operated by Government of Canada. The results of the Wiperator study performed to evaluate efficacy of AHP wipes and QAC wipes for decontaminating EBOV/Mak-contaminated carriers are displayed in Table 2. The values shown represent the combined data from three trials (three replicates each) utilizing the same lot of the AHP active, and one trial each (three replicates per trial) for three lots of the QAC active.

#### Negative Control Wipes (DMEM-Impregnated J-Cloth)

Following wiping of the EBOV/Mak-inoculated carrier with the DMEM-impregnated J-Cloth, infectious EBOV/Mak was still recovered from the inoculated carrier (Table 2). The recovered EBOV/Mak titers from these carriers ranged from ~4.1 to ~3.1 log_10_ TCID_50_/mL for the 5, 15, 30, and 60 s wiping times from an initial titer of ~6.6 log_10_ TCID_50_/mL. The log_10_ removal of EBOV/Mak from the inoculated carrier for each wiping time ranged from ~2.5 to ~3.5 log_10_, with a minimal increase in removal observed with increasing wiping time (Table 2). These values equate to removal of ~99.7% of virus from the inoculated carrier after 5 s wiping, vs. ~99.97% after 60 s wiping. The reduction in virus recovery from the inoculated carrier was attributed to removal rather than inactivation, since infectious virus was recovered from the secondary carriers following the transfer step.

In the transfer step, we examined the potential for transfer of virus removed from the inoculated carrier (and suspended within the J-Cloth wipe) to a secondary carrier. The EBOV/Mak titers recovered from the secondary carrier were found to range from ~3.9 to ~2.3 log_10_ TCID_50_/mL following transfer from the inoculated carriers wiped for 5, 15, 30, and 60 s. These values equate to transfer to the secondary carrier of ~0.41 to ~0.01% of the virus recovered from the inoculated carriers. These results indicate that the J-Cloth wipes impregnated with DMEM removed EBOV/Mak from the original contaminated surface, but also transferred a portion of the infectious virus to the secondary surface.

#### AHP-Impregnated Wipes

Following wiping of the EBOV/Mak-inoculated carrier with the AHP wipe, a low titer of infectious EBOV/Mak (mean values ranging from ~1.1 to ~0.20 log_10_ TCID_50_/mL; *n* = 3 replicates per time point) was recovered from the inoculated carrier after each wiping time (Table 2). The numbers of replicates yielding a positive viral detection were 5 out of 9, 2 out of 9, 2 out of 9, and 1 out of 9 for the 5, 15, 30, and 60 s wiping times, respectively.

In the transfer step, we examined the potential for transfer of virus removed from the inoculated carrier (and suspended within the AHP wipe) to a secondary carrier. The infectious EBOV/Mak titers recovered from the secondary carriers were ~0.9 log_10_ TCID_50_/mL and ~0.1 log_10_ TCID_50_/mL; *n* = 9 replicates) following transfer from the inoculated carriers wiped for 5 and 30 s, respectively. The virus was detected in 3 replicates out of the 9 evaluated from the inoculated carriers wiped for 5 s and in a single replicate from the inoculated carriers wiped for 30 s. No infectious EBOV/Mak was transferred from the inoculated carriers wiped for 15 or 60 s. These results indicate that the AHP wipe inactivated residual infectious EBOV/Mak virus that was removed during the wiping of the inoculated carriers at these times.

#### QAC-Impregnated Wipes

Following wiping of the EBOV/Mak-inoculated carrier with the QAC wipe, a low titer of infectious EBOV/Mak (mean ~0.6 log_10_ TCID_50_/mL; *n* = 9 replicates) was recovered from three inoculated carriers out of the total of nine replicates evaluated after 5 s wiping time (Table 2). One replicate out of nine tested after 30 s wiping time also yielded recoverable virus (for an overall mean of 0.2 log_10_ TCID_50_/mL, n = 9 replicates). After 15 and 60 s wiping, no infectious virus was recovered from the inoculated carriers.

In the transfer step, we examined the potential for transfer of virus removed from the inoculated carrier (and suspended within the QAC wipe) to a secondary carrier. A low titer of infectious EBOV/Mak (mean ~0.2 log_10_ TCID_50_/mL; *n* = 9 replicates) was transferred from the inoculated carrier wiped for 5 s (from one replicate out of the nine evaluated). No infectious EBOV/Mak was recovered from the inoculated carriers wiped for 15, 30, or 60 s. These results indicate that the QAC wipe inactivated EBOV/Mak virus that was removed during the wiping of the inoculated carriers.

### Results for VSV

The results of the Wiperator study performed to evaluate the efficacy of AHP and QAC wipes for decontaminating VSV-inoculated carriers are displayed in Table 3. The values shown represent the combined data from three trials (three replicates each) utilizing the same lot of the AHP active, and one trial each (three replicates per trial) for three lots of the QAC active.

#### Negative Control Wipes (DMEM-Impregnated J-Cloth)

Following wiping of the VSV-inoculated carrier with the DMEM-impregnated J-Cloth, infectious VSV was still recovered from the inoculated carrier after up to 60 s wiping time (Table 3). The viral inoculum averaged ~5.8 log_10_ TCID_50_/mL. The recovered VSV titers from the inoculated carrier ranged from ~3.9 to ~3.3 log_10_ TCID_50_/mL for the 5, 15, 30, and 60 s wiping times. The log_10_ removal of VSV from the inoculated carrier for each wiping time ranged from ~1.9 to ~2.5 log_10_ (Table 3). These values equate to removal of ~98.8% of virus from the inoculated carrier after 5 s wiping, vs. ~99.7% after 60 s wiping. The reduction in virus recovery from the inoculated carrier was attributed to removal rather than inactivation, since virus was observed on the secondary carriers following the transfer step.

In the transfer step, we examined the potential for transfer of virus removed from the inoculated carrier (and suspended within the J-Cloth wipe) to a secondary carrier. The VSV titers recovered from the secondary carrier were found to range from ~3.8 to ~3.3 log_10_ TCID_50_/mL following transfer from the inoculated carriers wiped for 5, 15, 30, and 60 s. These values equate to transfer to the secondary carrier of ~1.7% to ~0.4% of the virus recovered from the inoculated carriers. As was observed in the case of EBOV/Mak contaminated surfaces, this indicates that the J-Cloth wipes removed VSV from the original contaminated surface, although a portion of infectious virus was transferred to the secondary surface following use of these wipes.

#### AHP-Impregnated Wipes

Following wiping of the VSV-inoculated carrier with the AHP wipe, no infectious VSV was recovered from the inoculated carrier after the 5, 15, 30, or 60 s wiping times (Table 3). This indicates that the AHP wipe removed or inactivated essentially all of the VSV deposited on the original contaminated surface (~ 6.2 log_10_ TCID_50_/mL, estimated on the basis of the value for the dried positive control carrier).

Since no infectious VSV was recovered from the secondary carriers, it is presumed that any VSV removed from the inoculated carriers was inactivated during the transfer process or subsequent to being transferred onto the secondary carriers.

#### QAC-Impregnated Wipes

Following wiping of the VSV-inoculated carrier with the QAC wipe, no infectious VSV was recovered from the inoculated carrier after 5, 15, 30, or 60 s wiping time (Table 3). This indicates that the QAC wipe removed or inactivated essentially all of the VSV deposited on the original contaminated surface (~ 6.0 log_10_ TCID_50_/mL, estimated on the basis of the value for the dried positive control carrier).

Since no infectious VSV was recovered from the secondary carriers, it is presumed that any VSV removed from the inoculated carriers was inactivated during the transfer process or subsequent to being transferred onto the secondary carriers.

## Discussion

Disposable disinfectant pre-soaked wipes (DPW) are commonly used for reducing pathogen loads on HITES in healthcare and home settings (1, 2). These single-use wipes have the convenience of incorporating a microbicide, which offers the possibility both of removal of pathogens from a surface as well as inactivation of pathogens remaining on the wiped surface and within the wipe itself. The mechanical removal function of an impregnated wipe is not expected to be as great as that of a dry wipe, since DPW containing microbicides are pre-wetted and therefore have limited liquid absorbing capacity. Removal is facilitated by dilution of recovered virus within the fabric of the wipe and within the liquid used to impregnate the wipe. The inactivating function is determined by the efficacy of the incorporated microbicidal active for inactivating the pathogen of interest. This inactivation may occur within the wipe itself or within liquid expressed from the wipe during wiping of the original surface or a secondary surface.

The dual functionality of DPW has been referred to as *decontamination* by Sattar and Maillard (3) to reflect both the removal function and the inactivation function of such wipes. It has been apparent for some time that a wipe which only provides physical removal of a pathogen represents a potential source of transfer of the pathogen from a contaminated surface to a non-contaminated (secondary) surface during the wiping action (3, 5). In the case of emerging viruses, such as hemorrhagic fever-causing viruses, a low level of virus transferred from a contaminated surface to a secondary surface represents an undesirable vehicle for spread of infection to otherwise healthy individuals. This is particularly of concern for the Ebola virus, due to its low infectious dose (1–10 infectious units) (13, 22).

How significant is the potential for pathogen spread using wipes without appropriate microbicidal active ingredients? Until recently, it could only be surmised that spread of pathogens could occur through use of wipes without pathogen-inactivating activity. It has not previously been possible to quantitate and parse out removal and transfer of pathogens from one surface to another during the wiping process. Fortunately, in 2015 two methods were codified for assessing the abilities of DPW to decontaminate surfaces. These have been standardized as ASTM 2967-15 (21) in the USA and EN 16615 (11) in Europe. There are a few notable differences in the methodologies described in the two standards. For instance, EN 16615 uses a different soil load (0.3 g/L bovine serum albumin, vs. the tripartite soil load (21) described in the Methods section). The EN standard requires 5 pounds (2.5 kg) of pressure be applied to the wipe during the wiping operation vs. 0.33 pounds (0.15 kg) in the ASTM standard (6, 8). The wiping per EN 16615 is manually performed by laterally wiping with a cloth 2 times (forward and back) with a typical 5-min incubation after wiping. The Wiperator performs the wiping using a timed orbital action per ASTM 2967-15. EN 16615 utilizes a swab to sample the surface, while the entire carrier disk in the ASTM 2967-15 method is placed into a well-containing 1 ml of neutralizing agent for extraction. As a result, in the ASTM 2967-15 method 100% of the carrier surface is sampled after the wipe/transfer procedure instead of relying on swabs to sample a portion of the surface. The efficiency of swabbing for recovery of virus from a surface as done in the EN standard may be variable, depending on the surface used, the microorganism under evaluation, and the swab composition itself. In summary, both standards allow for estimation of extent of transfer of pathogen from an inoculated surface to secondary surfaces and each has characteristics that could be considered advantageous or disadvantageous.

A few publications have described the use of ASTM-2967-15 and the Wiperator instrument for evaluating the removal, transfer, and inactivation of bacterial pathogens by DPW (7, 8). These studies demonstrated that the potential for transfer of bacteria from the inoculated surface to one or more secondary (clean) surfaces depended both on the DPW used, as well as the bacterial species evaluated. For instance, several of the DPW tested were found to transfer *S. aureus*, while the potential of DPW to transfer *A. baumannii* was less, as was the extent of transfer of this microorganism. *Clostridium difficile* was transferred by all of the DPW wipes evaluated, and at the highest extent of the three microorganisms evaluated (7). These differences in potential for transfer likely reflect differences in the relative susceptibility of the microbes to the active ingredients incorporated into the DPW, as spore-forming bacteria (*C. difficile*) are known to be relatively less susceptible to microbicides. Differences in surface composition of the differing microbes also may play a role in their ability to attach to a surface.

To our knowledge, the first use of ASTM-2967-15 to evaluate viral removal, transfer, and inactivation using DPW was reported by Wesgate and Maillard (9). That study evaluated the bacteriophage MS2 as a surrogate for small, non-enveloped mammalian viruses. The results indicated that varying extent of removal of MS2 was achieved by the DPW, and importantly, all but one of eight DPW types with differing microbicidal active ingredients transferred infectious MS2 to a secondary surface (9). The small, non-enveloped viruses are generally less susceptible to inactivation by microbicidal products (23), and it is not surprising that sufficient inactivation of the MS2 to prevent transfer to a new surface during wiping was not achieved by most of the DPW investigated. Becker et al. (10) evaluated four DPW for inactivating three non-enveloped viruses (adenovirus, simian virus 40, and murine norovirus) using the EN 16615 methodology. In that study, only a DPW containing a peracetic acid active was found to cause sufficient inactivation of the three viruses to prevent transfer to secondary surfaces during wiping. The DPW with QAC or 2-propanol as active ingredients were found to transfer one or more of the test viruses. This again likely reflects the relatively lesser susceptibility of non-enveloped viruses to certain microbicidal products (23).

In the present study, the ASTM-2967-15 method was used to evaluate the performance of DPW containing one of two actives, AHP and QAC, vs. a control wipe (J-Cloth impregnated with DMEM). The model viruses used (EBOV/Mak and VSV) were enveloped viruses that were expected to be readily inactivated by the two active formulations tested. The negative control wipe was employed to help parse the contributions of removal and inactivation, as the negative control wipe was expected to remove, but not inactivate the viruses. The results obtained using the negative control wipe in the present study might also be expected when a DPW impregnated with an active ingredient with limited efficacy for the target pathogen is used. An example of an inappropriate active might be a detergent-impregnated DWP used for decontaminating a surface containing a non-enveloped virus (e.g., human norovirus) load.

The results reported herein quantitatively demonstrate the reduction of EBOV/Mak and VSV (each suspended in tripartite organic load) from a stainless steel carrier surface by wiping action and, in the absence of sufficient inactivating activity, the transfer of infectious virus from the inoculated carrier to a secondary surface. In a real-world situation, the use of a wipe having no or limited microbicidal activity would therefore be expected to afford some removal, albeit at the price of simply moving a portion of the contaminant from one location on the surface to another (24). For Ebola virus, the removal of 99.97% (3.5 log_10_) of virus from a surface contaminated with over 6 log_10_ of virus is insufficient from a public health standpoint, considering the low infectious dose for this virus. In addition, the spread of even a low percentage (e.g., 0.42%, equating to ~8000 infectious units) of the Ebola virus from an inoculated surface to a new surface could represent a significant opportunity of transmitting Ebola virus disease.

On the other hand, the present results demonstrate that a wipe impregnated with a sufficient concentration of an appropriate microbicidal active (in this case exemplified by AHP or QAC), given sufficient contact time, will remove virus from and inactivate remaining virus on a contaminated surface. Transfer of infectious virus from the inoculated surface to a secondary surface also is limited by the inactivating activity of the DPW. In additional studies involving EBOV/Mak and VSV, we have obtained similar efficacy data for DPW containing a variety of microbicidal actives, including 70% ethanol, 1% sodium hypochlorite, and a product containing 5% of a dual QAC (Cutts et al., publication in preparation).

## Conclusion

As observed previously (1–3, 5, 7–10) with bacterial and viral pathogens, the effectiveness of DPW for decontaminating surfaces is dependent upon a number of factors. These include the specific pathogen in organic load contaminating the environmental surfaces, the presence of an appropriate microbicidal active in the DPW, and the contact time (the time over which the pathogen is in contact with the microbicide).

In summary, removal of EBOV/Mak and VSV using wipes was extensive in this study. In the absence of a sufficient concentration and contact time of an appropriate microbicidal active in DPW (such as the AHP- and QAC-based DPW tested), transfer of a low, albeit significant (from an infectious unit/infectious dose perspective), quantity of infectious virus from the inoculated surface to a secondary surface was observed. In the case of Ebola virus, it is essential that a DPW with an appropriate microbicidal active, following the appropriate contact time, be used to prevent unintended transfer of infectious virus to a clean secondary surface (as observed in negative control /J-Cloth). Otherwise, there exists the possibility of dissemination of Ebola virus and the associated risk of transmission of Ebola virus disease. Disinfectant wipes pre-soaked with appropriately formulated microbicidal actives should be useful during outbreaks of Ebola virus as well as other highly contagious emerging viruses such as SARS-CoV-2 now causing pandemic COVID-19.

## Data Availability Statement

The original contributions presented in the study are included in the article/Supplementary Material, further inquiries can be directed to the corresponding author/s.

## Author Contributions

MI, TC, and ST conceived and designed the experiments. RN and TC analyzed the data. RN, MI, JR, TC, CR, and SK each contributed to the writing of the manuscript. All authors reviewed and approved the manuscript.

## Conflict of Interest

JR and MI are employed by RB, who also provided partial funding support for this study. RN provided data analysis and manuscript preparation as a paid consultant employed by RMC Pharmaceutical Solutions, Inc. The remaining authors declare that the research was conducted in the absence of any commercial or financial relationships that could be construed as a potential conflict of interest.
